# Microfluidic Spun Alginate Hydrogel Microfibers and Their Application in Tissue Engineering

**DOI:** 10.3390/gels4020038

**Published:** 2018-04-23

**Authors:** Tao Sun, Xingfu Li, Qing Shi, Huaping Wang, Qiang Huang, Toshio Fukuda

**Affiliations:** Beijing Advanced Innovation Center for Intelligent Robots and Systems, Beijing Institute of Technology, 5 South Zhongguancun Street, Haidian District, Beijing 10081, China; ilixingfu@163.com (X.L.); shiqing@bit.edu.cn (Q.S.); wanghuaping@bit.edu.cn (H.W.); qhuang@bit.edu.cn (Q.H.); tofukuda@nifty.com (T.F.)

**Keywords:** microfluidic spinning, alginate hydrogel microfibers, 3D assembly, tissue engineering

## Abstract

Tissue engineering is focusing on processing tissue micro-structures for a variety of applications in cell biology and the “bottom-up” construction of artificial tissue. Over the last decade, microfluidic devices have provided novel tools for producing alginate hydrogel microfibers with various morphologies, structures, and compositions for cell cultivation. Moreover, microfluidic spun alginate microfibers are long, thin, and flexible, and these features facilitate higher-order assemblies for fabricating macroscopic cellular structures. In this paper, we present an overview of the microfluidic spinning principle of alginate hydrogel microfibers and their application as micro-scaffolds or scaffolding elements for 3D assembly in tissue engineering.

## 1. Introduction

Organ transplantation is a well-established therapy for patients suffering from organs failure or damage; however, the long donors-waiting process causes the death of a number of patients, because the source of human organs is extremely limited [1]. To solve such a problem, tissue engineering approaches are trying to create artificial tissues as functional substitutes of human organs. In vivo, multiple cells are spatially arranged with a defined distribution; therefore, replication of such cell arrangement in vitro is critical to achieving the tissue regeneration in vitro [2]. Biomaterials-based scaffolds containing cells take an important extracellular matrix (ECM)-mimicked role to facilitate tissue-like cells arrangement and can further guide cells spreading, proliferation, and differentiation to form artificial tissues with specific shapes. However, traditional scaffolds lack the ability to control the behavior of a single cell and to generate large-scale vascularized tissue [3]. Microfabrication technology is being developed to reduce the size of the scaffolds into micro/nano-scale for tissue regeneration. On the one hand, these micro/nanoscaffolds can be processed with specific topographies and co-culturing cellular patterns for controlling cell behaviors; on the other hand, they can be employed as cell-carriers to achieve precise spatial positioning for the bottom-up construction of the vascularized tissues [4]. 

A variety of hierarchical scaffolds with controlled morphology and porosity have been engineered by various micro/nanoscaffolds, including polyethylene glycol (PEG) blocks, microdroplets, and micro/nanofibers et al. [5,6,7]. Among these hierarchical scaffolds, fabricating fibrous structures is one promising component, since micro/nanomicrofibers as scaffolding elements enable the engineered scaffolds to provide physical, chemical, and biological cues to regulate cellular behaviors [8,9]. Electrospinning and microfluidic spinning are two main methods to process micro/nanofibers. Electrospinning produces nanofibers by solidifying a charged polymer jet, and the fabricated nanofibers can be collected together to form net-like structures with nano-scale fiber diameter, high porosity, and stable mechanical properties. The net-like structures have been taken as the promising scaffolds for various tissue applications, such as regeneration of bone, cartilage, muscle, and vessel blood, et al. [10]. Compared with a high DC voltage for spinning nanofibers, microfluidic spinning based on microchannels processed by micro-electromechanical system (MEMs) technology can provide a milder spinning condition, and some unique characters are simultaneously involved in the fabricated microfibers, including cell encapsulation; the spatiotemporal control of microfiber the shape, size, and composition; and manipulation for the single microfiber [11]. Moreover, alginate as the main spinning material has the advantage of rapidly and simply forming gels with relatively stable mechanical properties and biocompatibility [12]. Therefore, microfluidic-spun alginate microfibers are a promising micro-scaffold with great application potential in tissue engineering.

## 2. Microfluidic Spinning Method

Relative to fluidic gravity and inertia, the fluid viscosity and surface tension affect the fluid flow behavior in the microchannel. Such change facilitates the generation of laminar flow in a simple microchannel if the Reynolds number R_e_ is small [13]. Different solutions form multi-laminar flows in contact with one another, and ions rapidly exchange between different laminar flow. In general, microfluidic spinning methods are dependent on the exchange of Ca^2+^ ions between alginate flow and CaCl_2_ flow [14]. Alginate microfibers with different morphologies and functions can be spun by three kinds of flow dynamic systems according to the different structures of the microchannels. 

### 2.1. Parallel Laminar Flows

**Figure 1:**
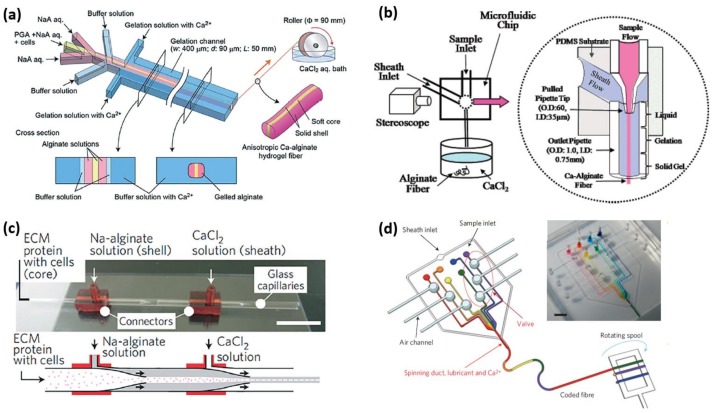
Microfluidic spinning system. (**a**) Parallel laminar flows [16]. Adapted with permission from [16]. Copyright 2012 Royal Society of Chemistry. (**b**) Coaxial laminar flows [21]. Adapted with permission from [21]. Copyright 2007 American Chemical Society. (**c**) Multi-coaxial microfluidic device [27]. Adapted with permission from [27]. Copyright 2013 Springer Nature. (**d**) Valve-involved spinning method [38]. Adapted with permission from [38]. Copyright 2011 Springer Nature.

Parallel laminar flows are generated in rectangular polydimethylsiloxane (PDMS) microchannel with a uniform thickness. The microchannel is fabricated by standard soft lithography and replica molding techniques, and its height is decided by the coating thickness of SU-8 [15]. Typical microfluidic device consists of three inlets and a long gelation microchannel, as shown in Figure 1a. Alginate solution is injected from the middle inlet by syringe pump, and then is squeezed by CaCl_2_ solution injected from two side inlets. Afterwards, sandwiched laminar flows are formed in the gelation microchannel. In laminar flows, Ca^2+^ ions in CaCl_2_ flow rapidly diffuse in the horizontal direction of the microchannel, and the alginate flow is gradually transformed into alginate microfibers. The cross-sectional shape of microfibers is a rounded rectangle, and the cross-sectional area can be modified by changing the flow rate of all solution [16]. Moreover, a thickener, such as dextran, can be added in CaCl_2_ solution to balance the viscosities of the solutions, which promotes the modification of the cross-sectional area. In addition, a buffer flow can be injected to form buffer flow between alginate and CaCl_2_ flows [17]. On the one hand, buffer flow moderates rapid cross-linking reaction to prevent the formation of gel irregular from blocking the microchannel; on the other hand, accelerating the buffer flow can terminate the spinning process to effectively pinch off the spun alginate microfibers from the gelation microchannel [18]. 

Alginate solution with different components or various concentrations can be simultaneously introduced to form a parallel multi-alginate flows in the gelation microchannel [19]. However, excessive inlets may waste the device space and expensive syringe pumps. Therefore, the multi-layered distribution channel networks connected to various inlets are employed to divide the different injected solutions into multi-flows and subsequently recombine flows with controlled array pattern [20]. Because of wide cross-section area of the recombined flows, the sufficient cross-linking reaction for solidifying the flows is difficult to achieve when dependent on CaCl_2_ flow. Therefore, the recombined flows are injected in a dish containing CaCl_2_ solution. In this case, the cross-sectional area of the spun microfibers is mainly decided by the size of the outlet of gelation microchannel rather than flow rate of the solutions. 

### 2.2. Coaxial Laminar Flows

The formation of coaxial laminar flows requires a microfluidic device with coaxial geometry [21]. The coaxial microfluidic device typically consists of an inner tapered glass capillary and an outer cylindrical or square tube, as shown in Figure 1b. Precise alignment and fixing technology is key to coaxially nesting the tapered part of inner capillary in the outer tube. Alginate solution is injected from inner capillary and then is sheathed by CaCl_2_ solution introduced from outer tube to form coaxial laminar flows. Hydrogel microfibers with circular or flat cross-sectional shapes can be spun responding to the cylindrical and square structure of the inlet, respectively [22,23]. Relative to the horizontal diffusion of Ca^2+^ ions in parallel laminar flows, the gelling direction in coaxial laminar flows is uniform in the cross-section of alginate flows. Furthermore, multi-layered coaxial laminar flows can be formed by adding the number of nested capillary [24]. Recently, a multi-coaxial microfluidic device has been widely constructed to generate a core-shell hydrogel microfiber, as shown in Figure 1c [25]. Different core parts of microfibers, including laminar flows of hyaluronic acid, biological cells, ECM protein, linear array of silk fibroin, and oil microdroplets can be sheathed by alginate hydrogel layer as outer protective shell [26,27,28,29,30]. Although such core-shell alginate microfibers can be formed in parallel laiminar flow, the encapsulation of the core parts is unstable [31]. In addition, coaxial flow enables the spun alginate microfiber to be spiraled by an unbalanced fluidic friction induced by its surrounding flow to form helical microfibers [32,33].

Besides glass capillary, PDMS microfluidic device can also be employed to generate a coaxial flow system. A step-like microchannel is characteristic of such PDMS device, which can be constructed by fabricating two consecutive SU-8 molds with different heights and replica molding. By aligning and bonding two step-like microchannel, a coaxial microchannel with different cross-section area can be fabricated. Alginate solution injected from the smaller microchannel can eject into CaCl_2_ flow injected from the microchannel with the bigger microchannel to form coaxial flow, and the flat alginate microfiber can be spun. In additions, the cylindrical microfibers can also be generated by employing the deflection of free-standing thin PDMS membranes to fabricate a cylindrical-flow PDMS channel [34]. Benefiting from the fabrication ease and stable physical properties of PDMS, sophisticated microchannel architecture can be constructed to involve some unique configuration characteristic into alginate microfibers. By engraving the grooved pattern on the inner surface of inlet, flat alginate fibers with grooves are spun under the sheath effect of CaCl_2_ flow [35]. Furthermore, a multiply stacked PDMS structure enables the formation of laminar flows in Z direction rather than in XY planar in traditional microchannel, and alginate microfibers with complex cross-sectional shapes can be formed by combining symmetric and coaxial laminar flows simultaneously formed in such microchannel [36]. 

### 2.3. Valve-Involved Spinning Method

Despite the fact that the above-mentioned laminar flow system enables mass production of alginate microfiber with various components, the distribution of these components in microfibers is decided by the injection position of alginate precursors in advance. For achieving the tunable distribution of components, a valve control system is designed to facilitate the programmable flow control in the spinning process [37]. Specifically, a parabolic PDMS membrane naturally generated in an air hole was installed on the inlet microchannel. Continuous air pressure or vacuum generated in air hole enables the membrane to be deflected or retrieved for ‘on-off’ switching of flow injection. By installing the valve on each inlet channel and independently controlling it, alginate microfiber with coded compositions can be spun, as shown in Figure 1d. Furthermore, the valve can be operated to control the core of droplets to be distributed either periodically or uniformly throughout the core-shell alginate microfiber [38].

In conclusion, fabricating alginate microfibers with complex compositions and morphology is a hotspot in microfluidic spun method. The complexity of the spun microfiber matches with the microchannel architecture. Rectangular PDMS microchannel is easily fabricated, in which parallel laminar flows can be formed to spun flat microfibers. Although the fabrication of glass microcapillaries requires skill, the generated coaxial flow can create novel core-shell cylindrical and helical microfibers. Furthermore, the most labor-intensive multi-layers PDMS microchannel with specific architectures allows for construct of alginate microfibers with tunable multi-compartments and morphologies. 

## 3. Alginate Hydrogel Microfibers as Scaffolds for Cell Culture

Laminar flows in microchannels can provide a mild aqueous environment to encapsulate cells into alginate microfibers with high viability, and the encapsulated cells can keep their activity for a long time, since alginate hydrogel provides a similar structure with the extracellular matrices in tissue. The easy encapsulation of cells and inherent biocompatibility enable microfluidic spun alginate microfibers to be widely applied as microscaffolds in tissue engineering.

### 3.1. Three-Dimensional Cell Culture

**Figure 2:**
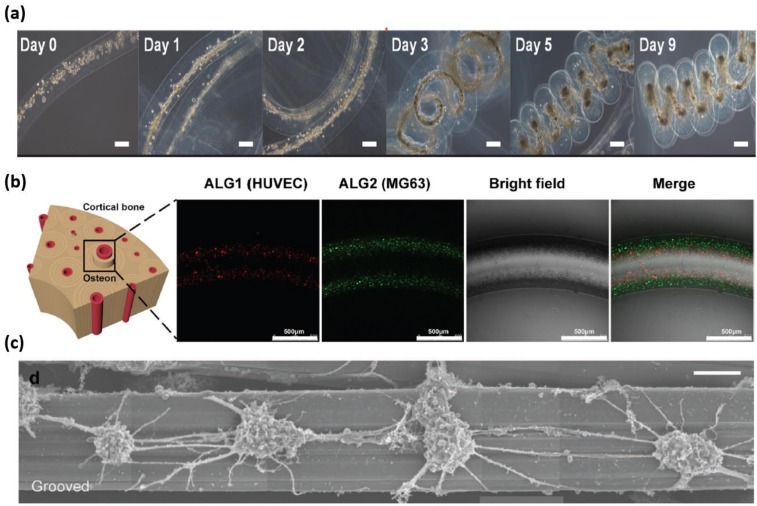
Cell-laden alginate hydrogel microfibers. (**a**) Self-assembly of de-differentiated fat cells-contained alginate microfiber [40], adapted with permission from [40]. Copyright 2015 Hsiao et al. Scale bar indicates 100 μm. (**b**) Osteon-like microfibers [43]. (**c**) Alginate microfiber with grooves for aligning nerve cells [35], adapted with permission from [35]. Copyright 2012 John Wiley and Son. Scale bar indicates 50 μm.

Since Ca^2+^ ions can generate a high degree of coordination with guluronate blocks of alginate chains, neighboring guluronate blocks of different alginate chains can then be combined to form “egg-box” junctions [12]. Plenty of junctions resemble a three-dimensionally networks to stably fix cells in the cross-linked alginate microfibers. Intrinsically higher porosity and larger pore sizes facilitate the exchange of nutrition and cell-secreted molecules and waste. However, due to the lack of adhesive sites on mammalian cell, pure alginate microfibers are usually employed as a simple mechanical support shell with function of cell immobilization and immunoprotection in vivo [21]. Utilizing the co-coaxial flows, Onoe et al. and Jun et al. and encapsulated the mixture of ECM proteins and pancreatic islet cells as cores into alginate microfibers as support shells [25,39]. ECM proteins can provide a suitable microenvironment to promote the islet cell to behave as they do in vivo. The alginate shell not only allows insulin secreted by islets cells to diffuse out of the fibers into the surrounding tissue, but also to pass and protect cells from immunological attack. In addition, differentiated fat cells encapsulated into such ECM proteins’ cores can successfully differentiate into smooth muscle-like cells, inducing the coiling of alginate microfibers, as shown in Figure 2a [40]. The circumferential orientation during cultivation is important for revealing the in vivo-like cell phenotype [41].

For promoting anchorage-dependent cells to interact with alginate hydrogel, other biopolymers have been incorporated with alginate to form composite hydrogel microfibers [12]. By the direct mixing of water-soluble chitosan solution and alginate solution, Lee et al. synthetized microfluidic-spun chitosan-alginate microfibers based on Ca^2+^ ions-induced cross-linked reaction, and the encapsulated human hepatocellular carcinoma (HepG2) cells were more viable than the cells encapsulated in pure alginate hydrogel [42]. Furthermore, the grafting of Arginylglycylaspartic acid (RGD) facilitates alginate hydrogel with higher biocompatibility for cell functional expression. Utilizing the combination of ionic and photoinitiated cross-linking, a RGD-incorporated double-layer of hollow microfibers containing human umbilical vascular endothelial cells (HUVECs) and human osteoblast-like cells (MG63) has been fabricated to form a biomimetic osteon-like structure. The encapsulated cells present an elongated cell morphology indicating excellent interactions between cells and surrounding matrix, which is difficult to achieve in chitosan-alginate microfiber. However, the introduction of Ca^2+^ ions and ionic cross-linking may reduce the mechanical properties of hydrogel microfibers [43]. As an alternative, alginate hydrogel has been regulated by incorporation of methacrylated gelatin (GelMA) containing RGD. Zuo et al. reported use glass microcapillary to transfer such composite materials into hydrogel microfibers with combination of high mechanical moduli and high biocompatibility [44]. 

### 3.2. Biomimetic Microorganoids

Because of the locating capacity of target fluids and cells on designed sections, multi-laminar flow enables alginate microfibers to be constructed into micro-organoids containing multiple cells with hierarchical structures mimicking nature microtissues, such as hepatic lobules, microvascular micro-scale blood vessel, and osteon. Because hepatocytes may lose their phenotype without the support of the feeder cells, in vitro patterned co-cultured microenvironment is important to maintain hepatocyte functions [45]. Utilizing three laminar flows generated in flat PDMS microchannel, Yamada et al. fabricated sandwiched Ba-alginate hydrogel microfibers, in which hepatocytes at the center are closely encased by Swiss 3T3 cells from two sides [19]. Compared with hepatocytes singly cultured in alginate hydrogel microfibers, hepatic functions including albumin secretion and urea synthesis are significantly enhanced due to such co-cultivation under high oxygen tension. Alginate lyase can enzymatically digest the surrounding hydrogel of the hepatic structure to obtain a micro-organoids mimicking in vivo hepatic cord structures without cells damage. Furthermore, they fabricated a stripe-patterned heterogeneous alginate microfiber with multi-sandwiched structure, which facilitates the relatively large-scale formation of rod-like heterotypic organoids for high retring of liver-specific functions [20]. 

Cylindrical microchannel can generate multi-layered coaxial laminar flows with circular cross-section. When the laminar flow at center is not involved in gelation reaction with the surrounding alginate flow containing cells, hollow alginate microfibers can be formed to mimic tubular microtissue in vivo [23]. By assembling such hollow alginate microfibers, Gao et al. fabricated large-scale 3D hydrogel structures with built-in microchannels, which enables the encapsulated L929 mouse fibroblasts to show higher cell viability relative to the hydrogel structures without built-in microchannels [46]. To further promote the bioactivity, Jia et al. employed a blend flow including gelatin methacryloyl (GelMA), alginate, and 4-arm poly (ethylene glycol)-tetra-acrylate (PEGTA) to form hollow alginate microfibers by successive ionic and photo crosslinking, and the resulting microfibers allow the spreading and proliferation of the encapsulated vascular cells, which is critical for creating functional vascular-like structure [47]. Furthermore, Xu et al. injected the rat blood into such hollow microfibers with helical structure to achieve their perfusabililty [48]. In additions, Wei et al. engineered hollow microfibers with double RGD-introduced alginate hydrogel layers. Osteocytes and human umbilical cord vein endothelial cells (HUVECs) were respectively encapsulated into inner and outer layer to mimic the structure of osteons, and such osteon-like microfibers enable cells to achieve the enhanced osteogenic and vasculogenic expression, as shown in Figure 2b [43]. 

### 3.3. Cell Guidance

For constructing linear tissue such as nerve bundles and muscle fibers, cells should be guided to achieve linear alignment during cultivation. The narrow and long morphology enables microfibers as a promising linear scaffold for regeneration of linear tissues. Yamada et al. fabricated solid-soft-solid flat microfibers by forming parallel alginate flows with and without propylene glycol alginate (PGA) [16]. Because of the physical restriction of two side solid region and the surrounding PLL layer for cell expanding, the neuron-like PC12 cells encapsulated in central soft region can be guided to elongate and generate cellular network along the microfiber direction. Furthermore, they designed a vertical micronozzle array structure to spin cylindrical alginate microfibers with eight soft regions uniformly located in the microfiber periphery [49]. Such complicated structure results in a higher ratio of linear colony formation of PC12 cells than the ratio in the proposed sandwiched structure, and the culture of PC12 cells in multi-soft regions facilitates the formation of one-millimeter-long intercellular networks that mimic nerve bundles in vivo.

Nerve cells can be alternatively cultured on the surface of alginate microfibers with high cell density, effective cell connection, and a good observation. Kang et al. fabricated grooved flat alginate microfibers to guide the neurites of neuron cells to form networks on the microfiber surface, as shown in Figure 2c [35]. The morphology of the connected neurite bundles of different neuron cells can be clearly observed. In contrast, most neuron cells aggregated on the edges of smooth microfibers, and the connection between different cells is difficult to be distinguished. The narrow groove bridges as a mechanical anchor are critical for guided cell expanding, and the same effect can be achieved by narrow microfibers with the nearly same size of the groove bridge. We used flat alginate microfibers (horizontal/vertical width < 40 µm) as scaffolding elements to guide fibroblast cells to form toroidal cellular micro-rings for the fabrication of microvascualr-like structure [50]. Compared with cells residing in microfibers, cells adhered on surface of alginate microfibers can be easily guided to form cellular layer with high cell density and uniform distribution. 

## 4. Alginate Hydrogel Microfibers as Scaffolding Elements for Higher-Order Tissue

Long and flexible structure and stable mechanical properties facilitate the excellent processability of alginate microfibers. The ease of mixing with other biomaterials promotes the high biocompatibility of the microfibers for cells growth. Microfluidic spinning method further involves turning biomimetic functions into microfibers. These features enable alginate microfibers as promising building blocks for the assembly of 3D macroscopic tissue-like structures. At present, the assembly strategy mainly consists of microfluidic printing and manipulation-based assembly. 

### 4.1. Microfluidic Printing

**Figure 3:**
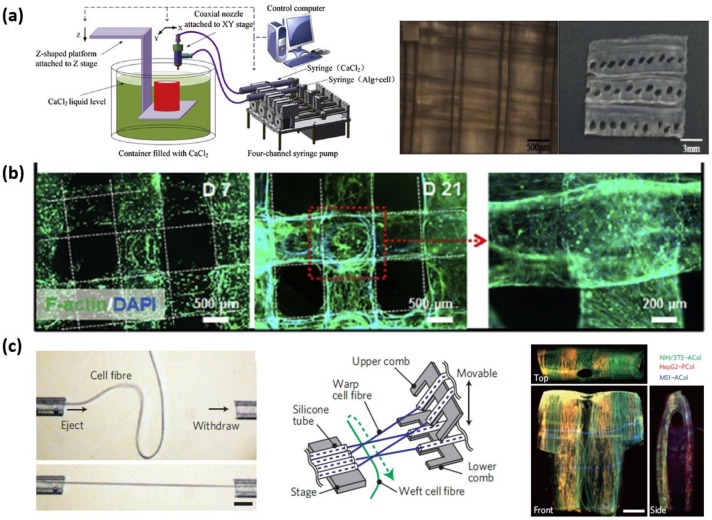
Higher-order assemblies for tissue engineering. (**a**) A printed cuboid structure consisting of 6 layers of hollow microfibers [46]. Adapted with permission from [46]. Copyright 2015 Elsevier Ltd. (**b**) Bioprinted hollow microfibers encapsulating vascular cells [47]. Adapted with permission from [47]. Copyright 2016 Elsevier Ltd. (**c**) Centimetre-scale woven macroscopic tissue [27], adapted with permission from [27]. Copyright 2013 Spring Nature. scale bars represent 1 mm.

In view of the capacity of the precise arrangement for cellular aggregates and cell-laden hydrogel microblocks, bioprinting becomes an efficient approach to directly construct the 3D cellular constructs with clinically relevant size, arbitrary shape, and vascularized architecture [51]. Compared with the pressure-induced generation of beads or lines of materials containing cells, alginate microfibers as printing ink can be generated in a relatively mild processing microenvironment. Ghorbanian, et al. employed a motorized stage to control the movement of microfluidic device for arranging the spun alginate microfiber [52]. When the movement speed is synchronized with the speed of fiber fabrication, cell-laden alginate microfibers can be smoothly deposited on the surface in the x-direction, and then in y-direction, and so on to form a multilayer net-like structure with high cell viability. Moreover, unique micro-structures generated by microfluidic spinning can be simultaneously involved into the generated net-like structure. Gao et al. offer a 3D bioprinting strategy based on hollow alginate microfiber spun by a coaxial nozzle as printing head [46]. With a Z-shape platform, the layer-by-layer fabrication can be achieved to form a cell-laden hydrogel structure with built-in microchannels, and cell culture can be perfused through the built-in microchannels showing the feasibility of nutrients flow, as shown in Figure 3a. Jia et al. improved the gelation materials form pure alginate into cell-responsive materials consisting of GelMA, alginate, and PEGTA [47]. The encapsulated endothelial and stem cells can spread and proliferate in the wall of the arranged hollow microfibers to form biomimetic built-in microchannels, leading to the formation of biological vasculature, as shown in Figure 3b. However, due to relatively higher mechanical properties, alginate microfibers are difficult to be arbitrarily arranged as the popular printing ink. Therefore, except for net-like structures, it is still a challenge to print other complicated structure mimicking natural tissue.

### 4.2. Guided-Assembly Method

The feasibility to handle single microfiber provides an alternative way to implement the higher-order assembly of alginate microfibers. With the guidance of weaving machine, weaving-based assembly of textile fibers can be widely applied in clothing industry. Long and flexible structure features enable alginate microfibers to have a potential to be weaved; however, new challenges to achieve such weaving assembly are generated due to the manipulation difficulty induced by micro-scale size, cell encapsulation, and weak mechanical properties relative to textile fibers. Onoe et al. developed a capillary-based “microfluidic handling” method to manipulate alginate microfibers [27]. The alginate microfiber sucked into capillary can be held by fluid resistance without any mechanical attachment. The clamped microfiber can move with the moving capillary and can be tensioned to form a bridge between two capillary holding two microfiber ends, respectively. Utilizing such bridge-shaped architecture, microfibers can be weaved to create a 3D net-like structure, and the net can keep stable by the friction among microfibers without secondary cross-linking reaction applied in microfluidic “printing” method, as shown in Figure 3c. The excess concentration of Ca^2+^ induced by secondary cross-linking reactions may induced cell death. When the medium is pushed out, the clamped end of the microfibers can be released from the capillary, and the fabric-like cellular structure can be obtained to mimic in vivo neuronal pathways in the brain.

Magnetic nanoparticles can be uniformly dispersed into alginate solution by ultrasonic vibration, and then be encapsulated into alginate microfibers by microfluidic spinning. The egg-box model of cross-linking enables the Ca-alginate gel to generate strong adhesive forces to capture the moving MNPs; therefore, magnetic alginate microfiber can be moved towards the external magnetic field. Based on such enhanced controllability, a series of magnetic guidance and no-contact manipulation strategies are developed for potential application in 3D cellular assembly. He et al. employed magnetic arrays to construct microfiber 2D letters and 3D textile-like patterns without the requirement of specific peripheral equipments [30]. The superparamagnetic properties of magnetic nanoparticles enable alginate microfibers to be stably adhered on the surface of the arrays, which is critical to keeping the patterned structure in the solution environments. We further miniatured the magnetic arrays by using an array of iron wires connected on the magnet surface. The iron wire can focus the magnetic field into the top of array, which facilitates more precise guidance for microfiber pattern relative to the magnet arrays [53]. Furthermore, magnetic response of alginate microfibers facilitates fabrication of more complicated structure using microfluidic “printing” method. We put a ring magnet under a support model (semi-sphere or curved semi-cylinder model) adhered on the bottom surface of dish filled with PBS. Because of the directional attraction of magnet, microfibers can neglect the effect of buoyancy to directly deposit on the surface of the models. Layer-by-layer microfibers can be assembled along the outline of the model with the movement of microfluidic device. After secondary cross-linking and removing the magnet, 3D cell-laden structure with the shapes of dome and curved arc can be obtained to mimic in vivo diaphragm beneath the lungs and curved vascular structure. 

## 5. Conclusions and Future Perspective

Microfluidic spinning method facilitates the production of alginate hydrogel microfibers with different morphologies and compositions to promote cell proliferation. Different cells, including fibroblast, endothelial cells, smooth muscle cells, nerve cells, hepatic cells, et al., have successfully encapsulated in or covered the alginate microfibers as microscaffolds for the research of cell biology and tissue engineering. However, due to the poor controllability of alginate microfibers, higher-order assembly is still challenging for the construction of macroscope cellular structure mimicking in vivo tissue. Therefore, the manipulation methods and assembly strategies should be further studied for construction of complex cellular structures.
